# Impact of poroelasticity of intraluminal thrombus on wall stress of abdominal aortic aneurysms

**DOI:** 10.1186/1475-925X-11-62

**Published:** 2012-08-29

**Authors:** Stanislav Polzer, T Christian Gasser, Bernd Markert, Jiri Bursa, Pavel Skacel

**Affiliations:** 1 Department of Solid Mechanics, Brno University of Technology, Technicka 2896/2, Brno, 616 69, Czech Republic; 2 Department of Solid Mechanics, The Royal Institute of Technology, Stockholm, Sweden; 3 Institute of Applied Mechanics (Civil Engineering), University of Stuttgart, Pfaffenwaldring 7, Stuttgart, 70569, Germany; 4 KTH Solid Mechanics, Osquars Backe 1, Stockholm, SE, 100 44, Sweden

**Keywords:** Pore pressure, Finite element analyses, Poroelasticity, Abdominal aortic aneurysm, Intraluminal thrombus

## Abstract

**Background:**

The predictions of stress fields in Abdominal Aortic Aneurysm (AAA) depend on constitutive descriptions of the aneurysm wall and the Intra-luminal Thrombus (ILT). ILT is a porous diluted structure (biphasic solid–fluid material) and its impact on AAA biomechanics is controversially discussed in the literature. Specifically, pressure measurements showed that the ILT cannot protect the wall from the arterial pressure, while other (numerical and experimental) studies showed that at the same time it reduces the stress in the wall.

**Method:**

To explore this phenomenon further a poroelastic description of the ILT was integrated in Finite Element (FE) Models of the AAA. The AAA model was loaded by a pressure step and a cyclic pressure wave and their transition into wall tension was investigated. To this end ILT’s permeability was varied within a microstructurally motivated range.

**Results:**

The two-phase model verified that the ILT transmits the entire mean arterial pressure to the wall while, at the same time, it significantly reduces the stress in the wall. The predicted mean stress in the AAA wall was insensitive to the permeability of the ILT and coincided with the results of AAA models using a single-phase ILT description.

**Conclusion:**

At steady state, the biphasic ILT behaves like a single-phase material in an AAA model. Consequently, computational efficient FE single-phase models, as they have been exclusively used in the past, accurately predict the wall stress in AAA models.

## Background

An abdominal aortic aneurysm (AAA) ruptures when the mechanical stress exceeds the local wall strength. Commonly used criterion of maximum diameter is not reliable enough to assess AAA rupture risk. It has been shown that some aneurysms with a diameter less than 5.5 cm rupture [1-4] and some large aneurysms do not. Therefore a Peak Wall Stress (PWS) [5-7] and Rupture potential index [8,9], also called Peak Wall Rupture Risk (PWRR) [7], have been found to be more reliable predictors of AAA rupture than the maximum diameter. However, PWS and PWRR depend on modelling assumptions; we are particularly concerned with constitutive descriptions of AAA tissues.

Intra-luminal Thrombus (ILT) is found in most AAAs of clinically relevant size [10] and its influence on wall stress remains controversial. ILT contains different types of blood [11,12] and other cells [13] as well as extracellular matrix (ECM) constituents. Specifically, new (fresh) ILT layers contain mainly fibrin fibres whereas the matured ILT, i.e. that in layers close to the vessel wall, shows a considerable amount of types I and III collagen [14]. Finally, the fibrous nano-structure is overlaid by a micro-structure of possibly interconnected cavities and pores filled by fluid [15-17]. The shape and dimension of these pores vary strongly with typical dimensions ranging from a few nanometres to micrometers [15]. Large pores may result from macrophages [11] and/or, especially in the abluminal layer, from micro-fissuring of the ILT. The ILT is a structural component with typical solid mechanical properties [18-22], enabling the load transition through the ILT fibrous network as mentioned by [23].

In-vivo [17] and in-vitro [24] experimental studies found that the pore pressure in the ILT is approximately equal to the blood pressure. Consequently, the ILT cannot reduce the pressure acting on the aneurysm wall and some authors [17] therefore concluded that the ILT cannot reduce wall stress either. Computational [25-28] and experimental [23] studies, with and without ILT, however demonstrate the opposite, i.e. that ILT considerably lowers stress and strain in the wall behind it. Regarding the ILT as a net of fibres that are connected to the aneurysm wall [23] could explain that the ILT lowers the wall stress even as the entire blood pressure penetrates through the pores towards the wall.

Different multi-phase and porous media theories used in modelling hydrated soft biological tissues have been proposed in literature [29-33]. Recently, even a porohyperelastic description of AAA (ILT and wall) has been proposed [34] using coupled porohyperelastic mass transport model [35]. However, the authors investigated a rather narrow range of ILT permeabilities ($9,1\cdot 10^{-11}\pm 0,54\cdot 10^{-11}{\text{m}}^{4}{\text{N}}^{-1}{\text{s}}^{-1}$) that unfortunately did not include the experimentally measured value [11] of $9,1\cdot 10^{-13}{\text{m}}^{4}{\text{N}}^{-1}{\text{s}}^{-1}$.

The present paper aims at investigating to what extent ILT’s poroelasticity influences wall stress predictions of AAA models. Specifically, the need for poroelastic modelling in the context of AAA wall stress assessment is studied, considering a wider interval of ILT permeability, as motivated by its microstructure.

## Methods

### Constitutive modelling of AAA tissues

#### Poroelastic description of the ILT

We consider the ILT as an elastic solid skeleton with a statistical distribution of interconnected pores (effective pores), which is fully saturated with a pore liquid. Consequently, the bulk volume *V* is given through the sum of the partial solid and fluid volumes, $V=V^{S}+V^{F}$, and the porosity (effective fluid volume fraction) is introduced as $\Phi =V^{F}/V$. Bounded intracellular/intrafibrillar fluids and sealed pores are considered as part of the solid phase and the ILT is modelled by Biot’s theory [36,37] at quasi-static conditions.

The solid displacement vector **u** and the pore-fluid pressure *p* are the primary unknowns governed by the momentum balance:

(1) $$0=div\left(\sigma_{E}-\alpha pI\right)+f$$

and the mass balance (continuity-like equation):

(2) $$\frac{\partial \zeta }{\partial t}+div\;q=0$$

where *div*(●) = *grad*(●)**I** denotes the divergence operator with the identity tensor **I**. The effective solid Cauchy stress tensor and Biot’s effective stress coefficient are denoted by **σ**_E_ and α, respectively, and ***f*** is the bulk body force per unit volume. Moreover, *ζ* describes the variation of fluid volume per reference volume and ***q*** is the flow flux vector, i. e. the fluid filter velocity. The model is completed by constitutive relations for the isotropic effective solid stress, described by the linear model

(3) $$\sigma_{E}=2\cdot G^{S}\;sym\left(gradu\right)+\left(K^{S}-\frac{2G^{S}}{3}\right)\left(divu\right)\cdot I$$

as justified from macroscopic properties of the ILT [20,38]. In order to account for finite strain computations, eq.(3) is implemented through the (objective) Jaumann stress rate [39].

In addition, the variation of the specific fluid volume:

(4) $$\zeta =\alpha \;div\;u+\frac{1}{M}p$$

and the fluid filter velocity vector:

(5) $$q=-k^{F}\left(\mathrm{grad}p-f^{F}\right)$$

were used with *M* denoting the (conventional) Biot modulus.

The effective Cauchy stress in the porous solid matrix is governed by an isotropic linear elasticity law with constant elastic tangent, where *G*^*S*^ and *K*^*S*^ denote the macroscopic shear and bulk moduli of the porous solid matrix. The fluid percolation is described by an isotropic Darcy-type filter law (5) with *k*^F^ being the apparent permeability in ${\text{m}}^{4}{\text{N}}^{-1}{\text{s}}^{-1}$ and ***f***^*F*^ the volume specific fluid body force. Note that tortuosity effects as well as anisotropic and deformation-dependent permeability changes [40] are not considered, and hence the present model aims at capturing only the salient features of ILT tissue. A more general description of hydrated soft tissue can be realized through the sequential solution of the porohyperelastic and mass transport problems, see [35] for example.

At physiological levels of pressure the solid and fluid constituents can be assumed as intrinsically incompressible, and the compressibility of the drained biphasic bulk material is magnitudes larger than that of its constituents. This simplifies the biphasic model, and a limit analysis gives [41] α = 1, *M* → ∞ and thus ζ≈divu, i.e. the fluid is only influenced by the solid volumetric strain. Neglecting (gravitational) body forces ($f=f^{F}=0$), the governing displacement–pressure (***u****p*) formulation of the two-phase model finally yields

(6) $$0=div\left(\sigma_{E}-pI\right)$$

(7) $$div\frac{\partial u}{\partial t}-k^{F}div\;grad\;p=0$$

such that the set {*G*^*S*^, *K*^*S*^, *k*^*F*^} of constitutive parameters defines ILT mechanical properties.

### Hyperelastic description of the wall

The AAA wall is regarded as a non-linear isotropic material. We neglected its porous properties due to its low permeability [42]$\left(k^{F}=7\cdot 10^{-16}m^{4}N^{-1}s^{-1}\right)$ compared to ILT. We modelled it by the strain energy function $\Psi =a_{1}\left(I_{1}-3\right)+a_{2}{\left(I_{1}-3\right)}^{2}$, which was originally proposed for rubber-like materials [43]. This model is frequently used for the AAA wall and $I_{1}=tr\;C$ denotes the first invariant of the right Cauchy Green deformation tensor $C=F^{\text{T}}F$, where **F** denotes the deformation gradient**.** Consequently, wall properties are characterized by the constitutive parameters {*α*_1_, *α*_2_}. In this study, the AAA wall is described by material parameters *a*_*1*_ *=* 174 kPa and *a*_*2*_ *=* 1.89 MPa reflecting mean population data from in-vitro testing of AAA tissue [44].

### Parameter identification

The present study considers that ILT’s shear modulus *G*^*S*^ gradually decreases from 21 kPa at the luminal site to 14 kPa at the abluminal site which refers to data published by Gasser et al. [20]. Likewise, ILT’s solid skeleton under tension is modelled as an incompressible material with a Poisson’s ratio of 0.4999.

Although the porosity *ϕ* of ILT tissue has not been measured directly, Φ=0.8 can be assumed based on the measured water content of 80% in ILT [18].

To estimate the apparent permeability $k^{F}=k^{S}/\mu$ the dynamic viscosity *μ* = 0.0044 Pa s of blood [45] is used, and the intrinsic permeability *k*^*S*^ in m^2^ is computed according to the model

(8) $$\frac{k^{S}}{a^{2}}=C_{1}{\left(\sqrt{\frac{1-\Phi_{c}}{1-\Phi }}-1\right)}^{C_{2}}$$

for fibrous materials [46,47]. Here, the percolation threshold is defined by the critical porosity $\Phi_{c}=0.0743$, above which permeating flow occurs; the dimensionless constants C_1_ = 0.491 and C_2_ = 2.31 are universal for fibre-based materials, i.e. they are independent from the actual micro-structural arrangement of the fibres. Specifically, these constants are valid for a wide range of materials like paper, wool, nylon, metal-fibre-based and collagen-fibre-based materials, when diluted by water, glycerol or air [47]. In equation (8) the characteristic length *a* defines the fibre radius of the fibrous material. ILT is regarded to be assembled by fibrin and collagen fibres, and considering radii from [16,48,49] 22 to 250 nm, the equation (8) defines a range of $7.5\cdot 10^{-14}<k^{F}<9.6\cdot 10^{-12}\;{\text{m}}^{4}{\text{N}}^{-1}{\text{s}}^{-1}$ for ILT apparent permeability.

The fibrous structure of ILT tissue is overlaid by a larger structure of pores, which, if interconnected, would define a network of large canaliculi and hence, these pores predominantly determine the ILT permeability. Considering characteristic lengths from [15] 1.0 μm to 20.0 μm for such a structure, model (8) enlarges the permeability spectrum, and consequently the present study considers a range of $7.5\cdot 10^{-14}<k^{F}<6.2\cdot 10^{-8}\;{\text{m}}^{4}{\text{N}}^{-1}{\text{s}}^{-1}$. It is emphasized that this wide range aims at accounting for inter- and intra-patient variabilities, and that the value $k^{F}=9.1\cdot 10^{-13}\;{\text{m}}^{4}{\text{N}}^{-1}{\text{s}}^{-1}$ as measured by an in-vitro experiment [11], is included.

### Mechanical AAA model

#### Geometry

**Figure 1:**
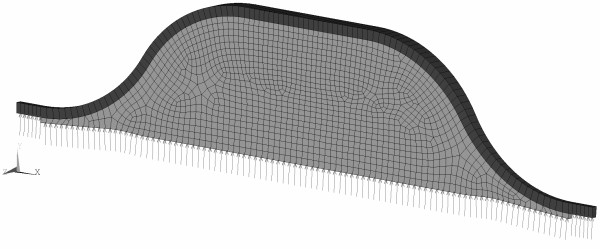
**Idealized AAA model to investigate the influence of the poroelastic properties of the ILT (shown in light grey) on the stress in the aneurysm wall (shown in dark grey).** The AAA model is loaded at the luminal surface by the blood pressure *p*_*b*_.

For simplicity and to obtain reviewable results, idealized axisymmetric AAA models were considered. Specifically, aneurysms with a length of 100 mm and maximum diameters of 60, 70 and 80 mm, subsequently denoted as AAA60, AAA70 and AAA80, were investigated. Likewise, a diameter of 20 mm describes the non-aneurysmatic infrarenal aorta, and a wall of a constant thickness of 1.8 mm was used. Bounded contact (to some extent motivated by an earlier study [23]) was selected between the ILT and the aneurysm wall. A typical investigated shape is shown in Figure 1.

The ILT was meshed by mixed tri-linear poroelastic elements, where the pore pressure is represented by an extra nodal degree of freedom, and a mixed tri-linear formulation was used for the aneurysm wall as programmed in the ANSYS 12.1., (ANSYS Inc. PA, USA) . The selected finite element formulations are suitable for incompressible deformations, i.e. provide locking-free results. It is mentioned that a single element across the aneurysm wall is used, and hence, a stress state that complies with the membrane theory is predicted. This assumption enforces an almost homogeneous tangential (membrane) stress across the wall without introducing residual stresses in the load-free configuration. Finally, it is noted that when using a single solid element across the aneurysm wall, the predicted radial wall stress is meaningless.

Mesh and time step convergence has been optimised to make sure that results will not be affected by these quantities by more than 5% as shown in Table 1. Chosen element size was then set to 1.5 mm and time step to $t_{L\max}=5.56\cdot 10^{-4}\text{s}$. Shorter time step would increase computational time too much while coarser mesh would induce error of more than 5%. The final mesh size is shown in Figure 1.

**Table 1:** Mesh size and time step sensitivity

**Case**	**Time step [s]**	**Element size [mm]**	**PWS [kPa]**	**Peak pore pressure [kPa]**	**Computational time [h]**
AAA80	$5.56\cdot 10^{-4}\;$	3	141.3	15.1	50
		1.5	141.1	15.8	60
AAA60	$5.56\cdot 10^{-4}\;$	1.5	142	14.8	30
	$2.27\cdot 10^{-4}\;$	1.5	142	15.1	60

Mesh sensitivity was tested for case AAA80, and time step convergence was studied for AAA60. Both models used an ILT permeability of *k*^*F*^ = 10^-12^ m^4^ N^-1^ s^-1^. When reducing the mesh size or the time step the results change negligible, and the computational time is inverse directly affected by the time step size.

### Boundary conditions

**Figure 2:**
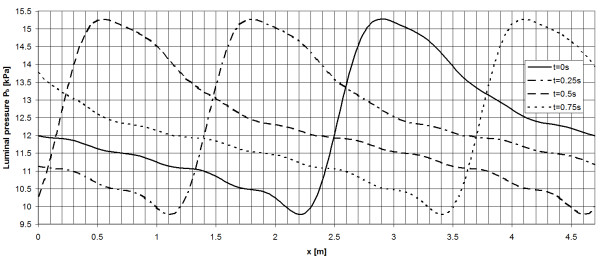
**Illustration of the pressure wave* p ***_***b***_*** (x,t) *applied to mimic in-vivo loading of the AAA.** The grid size of 100 mm reflects the axial dimension of the investigated AAA. The mean pressure of the prescribed pressure wave is defined by $p_{\mathrm{MEAN}}=\frac{1}{T}\;\int p\;dt=12.2\;\text{kPa}$, with T=1s denoting the time of the cardiac cycle.

Distal and proximal ends of the AAA are fixed and a blood pressure *p*_*b*_ = 12*kPα* is applied to the luminal surface and an initial value *p*_*b*_ = 12*kPα* of the pore pressure is prescribed within the entire ILT to reach faster convergence. Once the static solution was found, the pressure wave *p*_*b*_ = (x,t) was prescribed at the luminal surface as shown in Figure 2. Specifically, the pressure wave propagates along the axial coordinate *x* of the AAA as well as in time *t*. Similar pressure boundary conditions are frequently applied in Computational Fluid Dynamics (CFD) [50]. Specifically, we used a wave speed of 4.7 ms^-1^, which reflects the lower limit of experimental data [51], therefore any possible dynamic transient processes in the stress and pore pressure fields (i.e. pressure wave reflections and interference) of AAA will become more visible.

## Results

Transient analyses were carried out in ANSYS 12.1., (ANSYS Inc. PA, USA), which contains the above discussed non-linear material model for the aneurysm wall as well as poroelastic elements based on the Biot's theory [36] as introduced in the Method section. Using a 4CPUs, 12 GB RAM computer, the time needed for an analysis was up to 60 hours, depending on the particular model.

**Figure 3:**
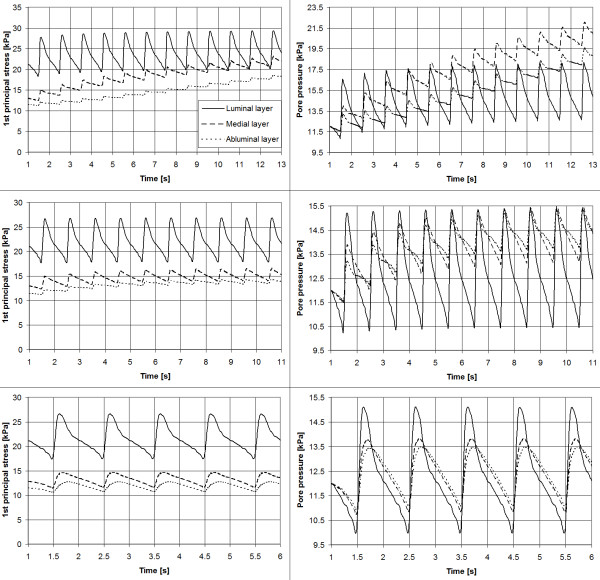
**Load transition processes in the AAA model AAA70 due to cycling blood pressure.** The analysis considers apparent permeabilities of $k^{F}=10^{-13}\;{\text{m}}^{4}{\text{N}}^{-1}{\text{s}}^{-1}$ (top row; steady-state solution not reached), $k^{F}=10^{-12}\;{\text{m}}^{4}{\text{N}}^{-1}{\text{s}}^{-1}$ (middle row) and $k^{F}=10^{-11}\;{\text{m}}^{4}{\text{N}}^{-1}{\text{s}}^{-1}$ (bottom row) of the ILT. Left column: Evolution in time of the first principal Cauchy stresses in the luminal, medial and abluminal layers of the ILT (legend is valid for all graphs). Right column: Evolution in time of the pore pressure in the luminal, medial and abluminal layers of the ILT.

For permeabilities of $k^{F}>10^{-13}\;{\text{m}}^{4}{\text{N}}^{-1}{\text{s}}^{-1}$_,_periodic steady state solutions were reached within 15 cardiac cycles, when the computation was terminated. The convergence of the principal Cauchy stresses in the aneurysm wall and of the pore pressure in the ILT of the aneurysm model AAA70 is illustrated in Figure 3. Note that at a fixed spatial coordinate, i.e. *x* = *x*_0_ the mean value of the steady-state pore pressure $p_{\mathrm{MEAN}}=\frac{1}{T}\;\int p\;dt=12.2\;\text{kPa}$ coincides with the prescribed mean blood pressure, and that the mean value of the steady-state stresses $\sigma_{i\;MEAN}=\frac{1}{T}\;\int \sigma_{i}\;dt;i=1,2,3$ matches the results of a traditional (single-phase) ILT description.

**Figure 4:**
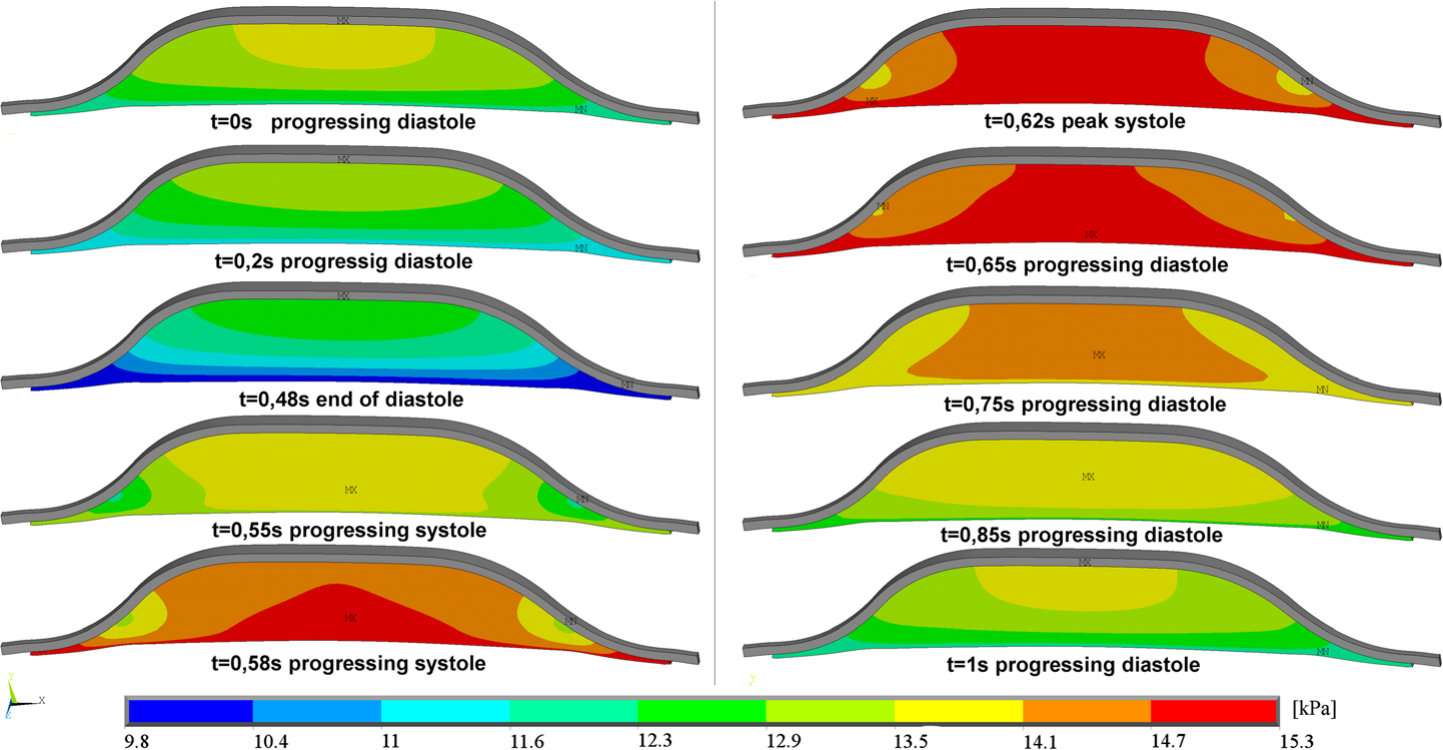
**Typical evolution of pore pressure during cardiac cycle; plotted for AAA60 and**$k^{F}=10^{-12}\;{\text{m}}^{4}{\text{N}}^{-1}{\text{s}}^{-1}$.

The pore pressure distribution during one cardiac cycle can be seen in Figure 4. It should be emphasized that the local pore pressure does not depend only on the distance from lumen but on the local geometry of AAA as well. (See neck areas in Figure 4). Finally, it is emphasized that the tensile stress in the wall during the systolic phase of the cardiac cycle is significantly reduced by the presence of the ILT, in comparison with the simulation neglecting the ILT as shown in Table 2. Table 2 shows that neither the material model of the ILT nor the prescribed BC affect resulting stresses. On the other hand, considering the ILT reduces stresses (PWS, maximum principal stress, local von Mises) significantly. The reduction is from 45% for AAA60 up to more than 60% for AAA80.

**Table 2:** Influence of material model and pressure boundary condition (BC) on stress in AAA

	**Peak Wall stress (PWS) [kPa]**			**Peak 1st principal stress [kPa]**		
**Material model of ILT**	**Without ILT**	**Single-phase**	**Poroelastic**	**Without ILT**	**Single-phase**	**Poroelastic**
Pressure BC	Constant	Constant	Pressure wave	Constant	Constant	Pressure wave
AAA60	259	143	142	289	153	152
AAA70	311	143	142	337	153	152
AAA80	375	141	141	400	149	149

Peak Wall Stress (PWS) (i.e. peak von Mises stress) and peak 1^st^ principal Cauchy stress in the AAA wall predicted by different Finite Element (FE) models. FE models either neglect the ILT or consider it using a single-phase or a poroelastic description. The pressure BC considers either a constant blood pressure (constant) or a time and space-dependent blood pressure wave (Pressure wave). Inertia effects are neglected in all cases.

**Figure 5:**
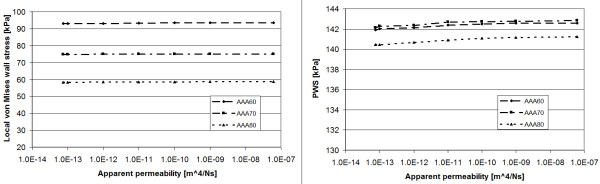
**Von Mises stress in the AAA wall as a function of the apparent permeability of the ILT at the systolic phase of the last calculated cardiac cycle.** Maximum wall stress in the middle of the aneurysm (left) and PWS (right).

From a clinical perspective the accurate prediction of PWS (and therefore of PWRR) are most important. PWS is defined as the maximum von Mises stress in the AAA wall, as usual, i.e. $PWS=Max\left[\frac{1}{\sqrt{2}}\sqrt{{\left(\sigma_{1}-\sigma_{2}\right)}^{2}+{\left(\sigma_{1}-\sigma_{3}\right)}^{2}+{\left(\sigma_{2}-\sigma_{3}\right)}^{2}}\right]$, and its dependency on the apparent permeability was investigated in greater detail. PWS occurred at the transition from the normal to the aneurysmatic aorta and does not depend on the ILT apparent permeability, see Figure 5(right). Similarly, the von Mises wall stress (and the principal stresses) in the middle of the aneurysm, i.e. behind the thick ILT, is independent from ILT apparent permeability; see Figure 5 (left).

Table 3 shows observed values of the pore pressure under the thickest ILT for various permeabilities. In the most extreme case, the Fluid pressure can decrease by 12% compared to the luminal pressure. Convergence was not reached for permeability values $k^{F}\leq 10^{-13}\;{\text{m}}^{4}{\text{N}}^{-1}{\text{s}}^{-1}$ within 15 cardiac cycles. For these permeabilities, pore pressure grew in time and exceeded the prescribed pressure by some 5kPa in 15^th^ cycle. It is expected, that converged pressure would be higher than 30kPa.

**Table 3:** Pore pressure underneath the thickest ILT [kPa]

$k^{F}\;\left[m^{4}N^{-1}s^{-1}\right]$	$7.5\cdot 10^{-14}\;$	**10**^**-13**^	**10**^**-12**^	**10**^**-11**^	**10**^**-10**^	**10**^**-9**^	$6.2\cdot 10^{-8}\;$
AAA60	22.4*	20.5*	14.8	13.9	15.2	15.3	15.3
AAA70	21.2*	19.6*	15.3	13.5	15.1	15.3	15.3
AAA80	18.9*	19.5*	15.8	13.9	15.0	15.3	15.3

Pore pressure underneath the thickest ILT for different AAA models and ILT permeabilities *k*^*F*^. *Steady state solution not reached after 15 cycles.

## Discussion

Clinical relevant AAAs frequently include ILT, whose impact on the biomechanical quantities is viewed with controversy in literature. FE models can help to understand how the blood pressure transforms into the wall tension; the present study broadens conventional (single-phase) AAA models [20,22,38] with a poroelastic description of the ILT. Although a poroelastic model reflects ILT micro structure closely, this approach has not yet been fully exploited and to the author’s knowledge, only a single work on this topic [34] has been reported in literature so far.

The present study found that at steady-state, stress in the AAA wall does not depend on the permeability of the ILT, and does not differ from single-phase ILT descriptions. This conclusion is of great importance, since a computationally much more efficient single-phase description can be used to predict the stress in the AAA wall reliably. Likewise, this finding validates earlier reported AAA models, which assumed ad-hoc a single-phase ILT. However, to study transport phenomena through AAA tissues, a poroelastic description can hardly be substituted.

The fact that poroelastic and single-phase descriptions of the ILT induce the same stress in the aneurysm wall may be explained by the particular nature of the structural problem. Specifically, the mobility of the pore fluid is constrained by the sealing of ILT outside (i.e. permeability magnitude of the aneurysm wall lower by 5 orders [42]) and the spatially constant blood pressure acting at the inside. These conditions lead to a pore fluid that is (almost) at rest (***q*** **≈ 0**) at the steady-state configuration, and hence, the single-phase and biphasic predictions coincide. Note that very low fluid filter velocities (10^-7^ ms^-1^) in the ILT have also been predicted with a permeable wall description [34] although this conflicts to some extent with in-vivo-measurements [52] showing a considerable change of ILT volume over the cardiac cycle. It can be explained by the fact that it is very difficult to reconstruct AAA geometry accurately and software currently work with the error of several percent which is comparable to the reported volumetric change [52]. We assumed intrinsically incompressible skeleton and pore fluid, such that for ***q*= 0** a macroscopic incompressible material is used. Therefore the volumetric part of the stress, which is affected by the fluid phase according to Biot’s theory [41], degenerates to a Lagrange contribution [53]. Similarly, the von Mises stress, which is independent from the hydrostatic pressure, cannot differ between poroelastic and single-phase descriptions.

Our numerical results demonstrate nicely that despite the existence of the ILT almost the entire blood pressure is transmitted to the wall. This was also confirmed by others [34] and explains why wall stress does not change whether the pressure is applied directly on the wall and or on the luminal surface of the ILT [54]. Most interestingly we showed that the ILT remarkably reduces the stress in the wall at the same time. This is in good agreement with both experimental data [17,23,24] and published numerical analyses [25,26,28,55]. Consequently, it integrates conflicting views regarding the role of the ILT in AAA biomechanics and demonstrates that the ILT helps to carry the load, in spite of the wall being exposed to the entire mean blood pressure. Specifically, for the investigated AAA models the ILT reduced wall tensile stress by a value between 46% (AAA60) and 62% (AAA80) relative to the model neglecting the ILT as shown in Table 2. It should be emphasised that this significant decrease in wall stress was obtained with a rather soft ILT [20], and using a stiffer ILT model [22,38] would reinforce the wall stress-diminishing effect [56] which explains why we obtained significantly higher wall stresses than the other study considering poroelastic description of the ILT [34]. On the other hand, it is noted that the predicted stress-diminishing effect is rather strong due to axisymmetry of the used model. For patient-specific geometries the PWS decrease due to the presence of the ILT varies from 5% to 59% [25,26].

Reported elastic ILT properties vary considerably [20-22,38] and changing them does not only influence the stress diminishing effect, but also the ILT poroelastic properties. We do not expect significant qualitative changes of our results to occur but a stiffer ILT would probably ensure that fewer loading cycles would be required to achieve a periodic solution.

Pore pressure under the ILT depended not only on the ILT permeability but also on the local geometry of the wall (see Figure 4) which may explain the differences in the reported in-vivo [17] and in-vitro [24] measurements, i.e. the measured values clearly depend on the position of pressure catheters.

Our study also found that a variation of the blood pressure along the AAA axis does not influence the stress predictions as shown in Table 2 and therefore the applied boundary condition, where the pressure changes in time and along the axis of the aneurysm, can be replaced by a pressure that varies only in time.

At cyclic loading the strain and pore pressure amplitudes in the abluminal ILT layer depend largely on the stiffness of the aneurysm wall. Our FE model used constitutive data for the AAA wall as reported in literature [57], where the stiffness at physiological deformation is about two times weaker than the more recently reported data [58]. While the predicted mean values of field variables might only be slightly affected by that discrepancy, their amplitudes could vary significantly.

For any kind of AAA model the interface conditions between wall and ILT define to a large extent the stress-diminishing effect of the ILT, and, following reported models in literature, we assumed a bounded (glued) interface. However, it is explicitly noted that opening the wall-ILT interface (cleavage formation between the ILT and the wall) allows the pore fluid to flow out, i.e. changes the exterior boundary condition for the fluid-phase at the interface.

Macroscopic ILT properties show an approximately linear First Piola Kirchhoff stretch response [15,22,38], however, the present study considers a linear response with respect to Cauchy stress, which underestimates the ILT stiffness at higher strains. As for any biological tissue, inter- and intra-patient variabilities of ILT are large, and it is not expected that this simplification could change the qualitative conclusions drawn by the present study. It should also be noted that more general numerical frames have been proposed [30,35], which allows considering additional features of highly deformable hydrated soft tissues.

We observed a significant pore pressure increase for low values of ILT permeabilities. It is noted that a comparable increase in pore pressure has not been reported from experimental studies, and the associated permeabilities may not be realistic. Moreover, the pore pressure also depends on the underlying poroelastic model and results might vary if alternative theories had been considered.

A poroelastic approach requires mechanical properties of a solid skeleton under drained conditions, which is difficult to obtain for biological tissue. Note that mechanical properties of biological tissues literately depend on moisture environment, and consequently all published properties of ILT relate to undrained conditions.

Our study considered an almost incompressible solid skeleton although any highly porous medium (like the ILT) behaves compressibly at least under compressive stresses. The incompressibility assumption aimed at capturing the tension properties of ILT, and hence may be applicable to tension-dominated stress state in an AAA. However, this assumption should be further investigated in (in-vitro) experimental studies to gain a better understanding of ILT properties at different loading conditions.

The ILT permeability might be inhomogeneous (e.g. due to its radially changing microstructure) and strain dependent [40], similarly to other soft biological tissues like articular cartilage [55]. Due to lack of experimental data we considered a constant permeability, which is thought to capture at least the most salient features of ILT tissue. Since this study demonstrated that wall stress predictions are insensitive to the permeability, its conclusions are not expected to change when refining the permeability model of the ILT.

## Conclusions

We have demonstrated in this study that including poroelastic description of the ILT in AAA does not change computed wall stress. Consequently we have back validated all studies using computationally more efficient single-phase material model for ILT. Our results also support the hypothesis stated by Thubrikar et al.[21] that ILT helps the wall to carry the blood pressure although it allows blood pressure acts on it. Finally our study helps to explain differences between in-vivo and in-vitro measurements by showing that pressure under ILT depends on the local geometry of the AAA.

## Competing interests

The authors declare that they have no competing interests.

## Authors’ contributions

SP carried out the FE calculations and helped to draft the manuscript. TCG participated in the design of computations and drafted the manuscript. BM carried out the theoretical description of used method and helped to draft the manuscript. JB helped to draft and critically revised the manuscript. PS helped with the FE calculations and result interpretation. All authors read and approved the final manuscript.
